# Prediction of Somatotype from Bioimpedance Analysis in Elite Youth Soccer Players

**DOI:** 10.3390/ijerph17218176

**Published:** 2020-11-05

**Authors:** Francesco Campa, Catarina N. Matias, Pantelis T. Nikolaidis, Henry Lukaski, Jacopo Talluri, Stefania Toselli

**Affiliations:** 1 Department for Life Quality Studies, University of Bologna, 47921 Rimini, Italy; francesco.campa3@unibo.it; 2 Faculdade de Educação Física e Desporto, Universidade Lusófona, 1749-024 Lisboa, Portugal; katarina_matias@hotmail.com; 3 CIPER—Interdisciplinary Center for the Study of Human Performance, Faculty Human Kinetics, University of Lisbon, 1495-751 Lisboa, Portugal; 4 Bioperformance & Nutrition Research Unit, Ingrediente Métrico S.A., 2740-262 Lisbon, Portugal; 5 School of Health and Caring Sciences, University of West Attica, 12243 Athens, Greece; 6 Department of Kinesiology and Public Health Education, Hyslop Sports Center, University of North Dakota, Grand Forks, ND 58202, USA; henry.lukaski@und.edu; 7 Department of clinical research and development, Akern Ltd., 56121 Pisa, Italy; Jacopo.talluri@akern.com; 8 Department of Biomedical and Neuromotor Sciences, University of Bologna, 40126 Bologna, Italy; Stefania.toselli@unibo.it

**Keywords:** anthropometry, BIA, body composition, football, predictive equation

## Abstract

The accurate body composition assessment comprises several variables, causing it to be a time consuming evaluation as well as requiring different and sometimes costly measurement instruments. The aim of this study was to develop new equations for the somatotype prediction, reducing the number of normal measurements required by the Heath and Carter approach. A group of 173 male soccer players (age, 13.6 ± 2.2 years, mean ± standard deviation; body mass index, BMI, 19.9 ± 2.5 kg/m^2^), members of the academy of a professional Italian soccer team participating in the first division (Serie A), participated in this study. Bioelectrical impedance analysis (BIA) was performed using the single frequency of 50 kHz and fat-free mass (FFM) was calculated using a BIA specific, impedance based equation. Somatotype components were estimated according to the Heath-Carter method. The participants were randomly split into development (*n* = 117) and validation groups (*n* = 56). New anthropometric and BIA based models were developed (endomorphy = −1.953 − 0.011 × stature^2^/resistance + 0.135 × BMI + 0.232 × triceps skinfold, R^2^ = 0.86, SEE = 0.28; mesomorphy = 6.848 + 0.138 × phase angle + 0.232 × contracted arm circumference + 0.166 × calf circumference − 0.093 × stature, R^2^ = 0.87, SEE = 0.40; ectomorphy = −5.592 − 38.237 × FFM/stature + 0.123 × stature, R^2^ = 0.86, SEE = 0.37). Cross validation revealed R^2^ of 0.84, 0.80, and 0.87 for endomorphy, mesomorphy, and ectomorphy, respectively. The new proposed equations allow for the integration of the somatotype assessment into BIA, reducing the number of collected measurements, the instruments used, and the time normally required to obtain a complete body composition analysis.

## 1. Introduction

Young soccer players are one of the most studied populations in sports science. In fact, there is a tremendous number of studies focusing on the search for talent and on the development of new techniques for evaluating physical features and their improvement in order to achieve high level performance [1,2]. Variables among the most informative and associated with physical performance are those related to body composition (BC). One of the most used techniques to evaluate BC in soccer players is bioelectrical impedance analysis (BIA) [2,3,4]. BIA uses bioelectrical properties of tissues to estimate BC variables such as total body water (TBW) and the respective intra and extracellular compartment (ICW and ECW) [5], fat mass (FM), fat-free mass (FFM) [6], and lean soft tissue (LST) [7]. This allows for the evaluation of a wide range of BC variables in not only a short time, but also easily and with high reliability and reproducibility [8]. Furthermore, its high use in athletes has meant that in recent years, several equations were proposed and validated against the four-compartment model (4C), the gold standard for the BC assessment at the molecular level [5,6,7].

In addition to the molecular level of the BC, other parameters are also frequently scrutinized in sports. In particular, these parameters include the morphological characteristics which belong to the whole body level, the fifth organizational level of BC proposed by Wang and collaborators [9]. In this regard, endomorphy, mesomorphy, and ectomorphy represent the three morphological components of the somatotype and their different combinations allow for the classification of the athlete’s body shape into one of 13 different categories. The evaluation of the somatotype in addition to other variables of BC allows for a complete and highly informative examination of the physical profile of the athletes. In fact, the morphological characteristics are related to physical performance [1,10], as well as discriminating for the player position/role and competitive level. In particular, a dominance of the endomorphic component was associated with a worse ability in performing repeated sprints with change of direction and with a low quality in functional movement patterns [10]. Additionally, significant differences in endomorphic values were observed between elite and sub-elite soccer players [1,10]. Furthermore, Víctor Cárdenas-Fernández et al. [11], in a recent study, showed that the dominant somatotype of soccer players was meso-endomorphic in goalkeepers, central for external defenders, balanced ectomorph in central defenders, balanced mesomorph in the case of midfielders, and meso-ectomorph in forwards/extremes.

However, the evaluation of the somatotype according to the Heath and Carter method requires the measurement of 10 specific anthropometric dimensions by a qualified anthropometrist and dependence on the measurement instruments, including possible technical errors that mitigate the accuracy of the evaluations [12]. In fact, these measurements include four skinfold thickness (triceps, subscapular, supraspinal, and medial calf), two circumferences (contracted arm and calf), and two diameters (humerus and femur), for which three different measuring instruments are required [13]. Unfortunately, when measuring a large sample of athletes, time constraints limit the amount of measurements that can be taken using different tools and therefore, the best decision may be to conduct quick but precise evaluations that allow for the measurement of a sufficient number of variables. Reducing the number of instruments used and the measurements collected for the evaluation of the somatotype would permit the integration of the analysis of morphological characteristics to BIA, thus allowing the analysis of a large number of variables belonging to different levels of BC in a practical way and in a short amount of time. Therefore, the aim of this study is to generate new predictive models for the somatotype assessment in youth elite soccer players, including bioelectrical and their BC-derived parameters, and to reduce the number of anthropometric measurements already required by the Heath and Carter method.

## 2. Materials and Methods

### 2.1. Participants

173 soccer players (age, 13.6 ± 2.2 years, mean ± standard deviation; body mass index, BMI, 19.9 ± 2.5 kg/m^2^), from the under 10 to under 17 age categories, registered in a professional Italian soccer team participating in the first division (Serie A), were selected to participate in the study. The players voluntarily decided to participate and their parents provided informed consent after a detailed description of the study procedures. The project was conducted according to the Declaration of Helsinki and was approved by the Bioethics Committee of the University of Bologna (Approval Code: 25027).

### 2.2. Procedures

All anthropometric measurements were profiled by an accredited anthropometrist (S.T.). Height was measured to the nearest 0.1 cm using an anthropometer (GPM, DKSH, Zurich, Switzerland). Body weight was measured to the nearest 0.1 kg using a calibrated electronic scale. BMI was calculated as body mass in kilograms divided by the square of height in meters. Somatotype components were calculated according to the Heath-Carter method [13], for which girth was taken to the nearest 0.1 cm using a tape measure, breadth was measured to the nearest 0.1 cm using a sliding caliper, and skinfold thicknesses at four sites (triceps, subscapular, supraspinal, and medial calf) were measured to the nearest 0.1 mm using a Lange skinfold caliper. The raw impedance parameters, resistance (R), and reactance (Xc) were obtained with a bioimpedance analyzer (BIA 101 Anniversary; Akern Srl, Florence, Italy) at a frequency of 50 kHz, according to the standard procedures [14,15]. Phase angle (PhA) was calculated as the arctangent of Xc/R × 180°/π and FFM using a specific equation [6]:

FFM = − 2.261 + 0.327*stature^2^/R + 0.525*body weight + 5.462*1,

### 2.3. Statistical Analysis

Descriptive statistics were performed to characterize the sample. All variables were checked for normality, using the Kolmogorov-Smirnov test. Stratified random assignment based on age categories was used to assign participants to either a development group (*n* = 117) or a cross validation group (*n* = 56). Stepwise regression analysis was used to evaluate the ability of variables (R, Xc, PhA, FFM, FFM/stature, chronological age, stature, weight, BMI, skinfold thickness, circumferences, stature^2^/resistance, and stature^2^/reactance) to predict endomorphy, mesomorphy, and ectomorphy in the development group. During model development, normality of residuals and homogeneity of variance were tested. The criterion for inclusion of a predictor was significant at *p* ≤ 0.05. If more than one variable remained in the model, a variance inflation factor (VIF) for each independent variable was calculated and values below five were considered as not having multicollinearity [16]. To cross validate the developed models, the resulting equations were applied to the cross validation group according to the statistics method described elsewhere [16]. A paired sample t-test was used to compare the mean values obtained from the reference technique and from the new method. To assess the accuracy of the new predictive models, validation parameters included the analysis of the coefficient of determination and the pure error. The pure error was assessed using the following equation (∑(Y¨ − Y)^2^/n^1/2^, where Y¨ is the predicted variable, Y is the observed variable, and *n* is the number of participants [17]. Additionally, the concordance correlation coefficient (CCC) using the Lin approach [18] calculated with MedCalc Statistical Software v.11.1.1.0, 2009 (MedCalc, Mariakerke, Belgium) was performed. The CCC contains a measurement of precision and accuracy (*ρ*_c_ = *ρ C*_b_): where *ρ* is the Pearson correlation coefficient, which measures how far each observation deviates from the line of best-fit and is a measure of precision, and *C*_b_ is a bias correction factor that measures how far the best fit line deviates from the 45° line through the origin and is a measure of accuracy. Finally, agreement between the developed models and the reference procedure was assessed using the Bland-Altman method [19], including the analysis of the correlation between the mean and the difference of the methods and an estimate of the limits of agreement. Data were analyzed with IBM SPSS Statistics, version 24.0 (IBM Corp., Armonk, NY, USA).

## 3. Results

The participants’ characteristics for the development and cross validation groups are presented in Table 1.

### 3.1. Models Developments

Only variables contributing as significant predictors using a backward stepwise approach were used in the models’ developments. The final prediction models are shown in Table 2.

### 3.2. Cross Validation of Derived Prediction Models

A cross validation was performed and the results of the regression parameters, CCC, and agreement analyses between the somatotype components estimated from the new developed models and the reference procedure are presented in Table 3 (cross validation panel). 

**Figure 1:**
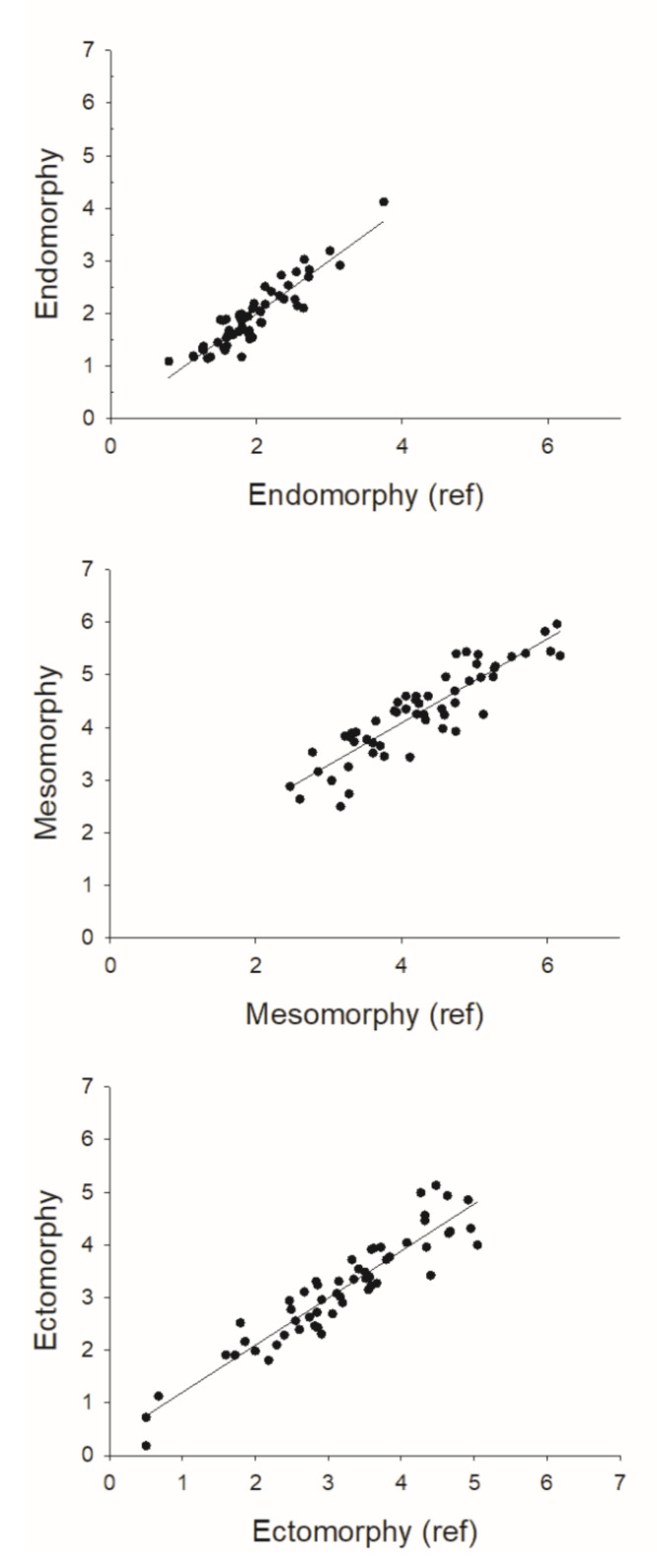
Scatterplot of the relationship between the somatotype components obtained by the reference method and the new formulas.

**Figure 2:**
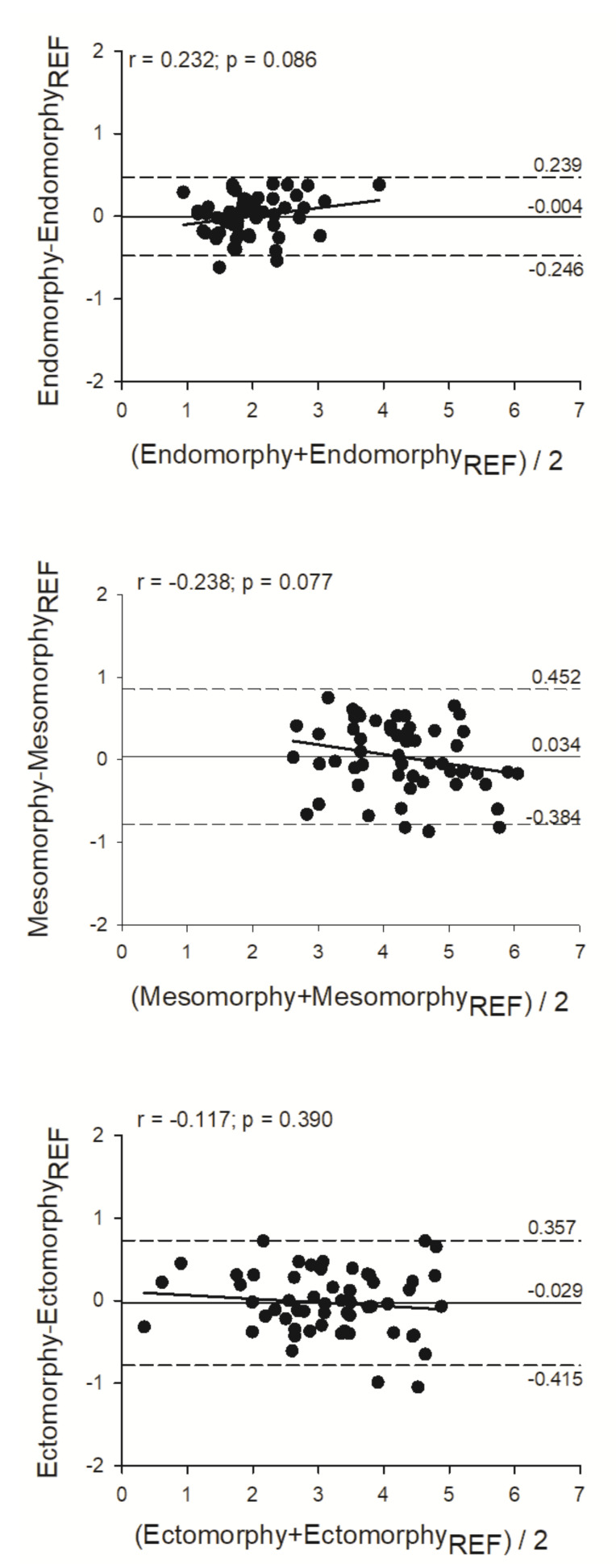
Bland-Altman analysis of the agreement between methods for the somatotype’s components estimation. The middle solid line represents the mean differences between the values obtained by the new equations and the reference method (Ref) and was predicted by the equations. The upper and lower dashed line represents the 95% limits of agreement (± 1.96 SD). The trend line represents the degree of association between the differences of the methods and the mean of both methods, as illustrated by the coefficient of correlation (r).

Regarding the paired sample t-test, no differences between methods were observed for any of the calculated somatotypes estimation (*p* > 0.05). Concerning the regression analysis, the methods were highly correlated (R^2^ ≥ 0.80; *p* < 0.001). The predictive models developed in phase I for the three somatotypes explained 84%, 80%, and 87% of the variability observed in the values of the reference methods for endomoorphy, mesomorphy, and ectomorphy, respectively. The precision and accuracy of the methods was higher than 0.89 and 0.99, respectively, with a CCC between the new method and the reference procedure superior to 0.89 (Table 3, Figure 1). From the agreement analysis, we observed no trend between the mean and the differences of the methods for any of the somatotypes and small limits of agreements (Figure 2). 

## 4. Discussion

The purpose of this study was to propose a new strategy for the somatotype assessment, which would allow one to reduce the time and number of measurements, as well as instruments used in the traditional Heath and Carter method. The new proposed equations have been developed considering anthropometric characteristics and bioimpedance parameters in order to be able to use these two techniques to obtain a large number of BC parameters in a short period of time. All the measures necessary for calculating the somatotype according to Heath and Carter have been inserted into the predictive equations’ development procedures of this study; nevertheless, only triceps skinfold thickness and the contracted arm and calf circumferences, as well as BMI and the impedance derived parameters were predictors of somatotype in youth soccer players.

Recently, Rudnevet al. [20] proposed a BIA based equation to estimate the three components of the somatotype in the general population. The same research group had already proposed two other equations, one for children and adolescents and the other for the elderly [21]. However, these studies only have a development group and not a validation group, so the accuracy of the models is not completely certain. Additionally, these equations may not be suitable for the sports population as they represent a special population. On the contrary, a cross validation was performed in this study and a very strong correlation was observed between the developed equations and the reference method (R^2^ ≥ 0.80). Moreover, precision and accuracy between the new predictive equations and the reference procedure were analyzed with concordance correlation coefficient analysis. In this regard, a moderate strength of agreement [22] between the methods was observed in estimating endomorphy and ectomorphy (CCC = 0.91 and 0.93, respectively), while for the mesomorphy, a weaker agreement was observed between the methods (CCC = 0.88). Furthermore, the magnitude of the differences between the new predictive model and the reference method was examined according to the Bland-Altman method [19]. Therefore, at an individual level, no bias between the mean and the differences of the methods for any of the somatotypes were observed and small limits of agreements are presented.

In addition to the classical BIA approach, the vector variant (BIVA) has also proved useful for the evaluation of the somatotype. Indeed, recent studies [23,24,25] have shown how raw bioimpedence parameters (R, Xc, and PhA) are able to discriminate the different somatotypes and are associated with the morphological components. In particular, Campa et al. [23] have shown that PhA and mesomorphy are positively correlated, while PhA and ectomorphy show an inverse association. This is because PhA accurately represents the ICW/ECW ratio [26,27] and this is superior in subjects with a marked muscularity typical of the mesomorphic constitution [28]; in fact, PhA fell into the predictive model of mesomorphy in this study.

The somatotype assessment represents a highly informative analysis for the BC of athletes. Mesomorphic component is linked to individual sports that require muscle strength, such as ball games [29], while ectomorphy is predominant in runners, especially those involved in long distance running [30]. Then, anthropometry and morphological features play a crucial role in determining potential success in sports [31]. Furthermore, being that soccer players vary in morphology according to the player’s position [1,11], somatotype assessment in young athletes can prove useful in game choices and talent prediction.

A strong point of this work is represented by the fact that in these new equations, the measurements required for the calculation of the somatotype are reduced, eliminating three adipose skinfold thicknesses (subscapular, supraspinal, and medial calf) out of four and two diameters (humerus and femur), thus avoiding the use of the bone caliper for their measurement. In this regard, although the evaluation of morphology is an important aspect, it is not often integrated into routine evaluations due to the numerous measurements and tools required, which can involve a high operator dependent error as well as long processing times [12].

Some limitations inherent in this study need to be mentioned. First of all, despite being a large sample, the new equations were developed and validated on a sample of young male soccer players, so the results of this study are not extendable to all young athletes, adults, or female athletes. Furthermore, BIA was performed using a single frequency and this does not guarantee the reproducibility of the results with different impedance devices [32]. On the other hand, a strength of the study is its novelty, considering the lack of research on the relationship of somatotype and BIA in young male soccer players. The findings would be of great practical importance for coaches and fitness trainers working with soccer players in terms of talent identification and players’ selection, also taking into account the somatic maturation of the athletes. In addition, since body composition is a major component of sport related physical fitness [33], our data might be used for training monitoring. 

## 5. Conclusions

BIA is widely used and often favored over other assessment methods in sports because it provides a wide range of BC parameters in an easy, fast, and low operator dependent manner. On the contrary, the evaluation of the morphological characteristics according to the Heath and Carter approach requires the use of different tools and the collection of many measurements that are often time consuming. The new proposed equations make it easier to integrate the somatotype assessment with BIA, thus obtaining a complete analysis of the BC in youth elite soccer players.

## Figures and Tables

**Table 1:** Descriptive Characteristics of the Development and Cross validation Groups.

	Development Group (*n* = 117)	Cross Validation Group (*n* = 56)
	Mean ± standard deviation	Mean ± standard deviation
Age (years)	13.5 ± 2.1	13.5 ± 2.3
Weight (kg)	53.5 ± 14.9	54.0 ± 14.9
Stature (cm)	162.3 ± 15.2	162.2 ± 16.7
Body mass index (kg/m^2^)	20.0 ± 2.5	19.9 ± 2.5
Resistance (ohm)	555.4 ± 89.7	537.9 ± 73.3
Reactance (ohm)	64.4 ± 6.9	62.1 ± 9.9
Phase angle (degree)	6.7 ± 0.9	6.7 ± 1.1
Fat free mass (kg)	47.6 ± 12.6	48.3 ± 12.5
Triceps skinfold (mm)	8.1 ± 2.6	7.6 ± 2.3
Subscapular skinfold (mm)	6.6 ± 2.1	6.3 ± 1.7
Supraspinal skinfold (mm)	5.8 ± 2.6	5.4 ± 1.9
Medial calf skinfold (mm)	6.9 ± 2.5	6.6 ± 2.4
Contracted arm circumference (cm)	25.5 ± 4.1	25.9 ± 3.6
Calf circumference (cm)	33.3 ± 4.8	33.8 ± 3.5
Humerus width (cm)	6.3 ± 0.6	6.3 ± 0.7
Femur width (cm)	9.1 ± 0.7	9.2 ± 0.7
Endomorphy	2.1 ± 0.7	1.9 ± 0.5
Mesomorphy	4.1 ± 1.1	4.3 ± 0.9
Ectomorphy	3.3 ± 1.0	3.1 ± 1.1

**Table 2:** Prediction Models for Endomorphy, Mesomorphy, and Ecthomorphy Based on Anthropometrics and Bioimpedance-Derived Variables.

	Predictors	R	R^2^	SEE	VIF	Prediction Equation
Endomorphy	S^2^/R BMI Triceps skinfold	0.92	0.86	0.28	4.22 3.61 1.55	y = −1.953 − 0.011 × S^2^/R + 0.135 × BMI + 0.232 × triceps skinfold
Mesomorphy	PhA CAC CC Stature	0.93	0.87	0.40	1.49 2.34 1.84 2.68	y = 6.848 + 0.138 × PhA + 0.232 × CAC + 0.166 × CC − 0.093 × stature
Ectomorphy	FFM/S Stature	0.93	0.86	0.37	4.75 4.75	y = −5.592 − 38.237 × FFM/S + 0.123 × Stature

Abbreviations: R = multiple correlation coefficient; R^2^ = multiple coefficient of determination; SEE = standard error of estimate; VIF = variation inflation factor; S^2^/R = stature^2^/resistance; BMI = body mass index; PhA = phase angle; CAC = contracted arm circumference; CC = calf circumference; FFM/S = fat-free mass/stature.

**Table 3:** Cross Validation of the Somatotype’s Predictive Models and the Reference Procedure.

	Regression Analysis		CCC Analysis			Agreement Analysis		
	R^2^	PE	CCC	ρ	C_b_	Bias	95% LoA	Trend
Cross Validation								
Endomorphy	0.84	0.222	0.92	0.9158	0.9954	−0.004	−0.246; 0.239	r = 0.232 (*p* = 0.086)
Mesomorphy	0.80	0.422	0.89	0.8920	0.9932	0.034	−0.034; 0.452	r = −0.238 (*p* = 0.077)
Ectomorphy	0.87	0.389	0.93	0.9335	0.9987	−0.029	−0.415; 0.357	r = −0.117 (*p* = 0.390)

Abbreviations: R^2^, coefficient of determination; PE, pure error; CCC, concordance correlation coefficient; ρ, precision; C_b_, accuracy; LoA, limits of agreement.

## References

[1] Slimani M., Nikolaidis P.T. (2019). Anthropometric and physiological characteristics of male soccer players according to their competitive level, playing position and age group: A systematic review. J. Sports Med. Phys. Fitness.

[2] Campa F., Silva A.M., Iannuzzi V., Mascherini G., Benedetti L., Toselli S. (2019). The role of somatic maturation on bioimpedance patterns and body composition in male elite youth soccer players. Int. J. Environ. Res. Public Health.

[3] Castizo-Olier J., Irurtia A., Jemni M., Carrasco-Marginet M., Fernández-García R., Rodríguez F.A. (2018). Bioelectrical impedance vector analysis (BIVA) in sport and exercise: Systematic review and future perspectives. PLoS ONE.

[4] Núñez F.J., Munguía-Izquierdo D., Suárez-Arrones L. (2020). Validity of Field Methods to Estimate Fat-Free Mass Changes Throughout the Season in Elite Youth Soccer Players. Front. Physiol..

[5] Matias C.N., Santos D.A., Júdice P.B., Magalhães J.P., Minderico C.S., Fields D.A., Lukaski H.C., Sardinha L.B., Silva A.M. (2016). Estimation of total body water and extracellular water with bioimpedance in athletes: A need for athlete-specific prediction models. Clin. Nutr..

[6] Matias C.N., Campa F., Santos D.A., Lukaski H.C., Sardinha L.B., Silva A.M. (2020). Fat-free Mass BIA Predictive Equation for Athletes Using a 4-Compartment Model. Int. J. Sports Med..

[7] Sardinha L.B., Correia I.R., Magalhães J.P., Júdice P.B., Silva A.M., Hetherington-Rauth M. (2020). Development and validation of BIA prediction equations of upper and lower limb lean soft tissue in athletes. Eur J. Clin. Nutr..

[8] Kyle U.G., Bosaeus I., De Lorenzo A.D., Deurenberg P., Elia M., Gomez J.M., Heitmann B.L., Kent-Smith L., Melchior J.C., Pirlich M. (2004). Bioelectrical impedance analysis-part I: Review of principles and methods. Clin. Nutr..

[9] Wang Z.M., Pierson R.N., Heymsfield S.B. (1992). The five-level model: A new approach to organizing body-composition research. Am. J. Clin. Nutr..

[10] Campa F., Semprini G., Júdice P.B., Messina G., Toselli S. (2019). Anthropometry, physical and movement features, and repeated-sprint ability in soccer players. Int. J. Sports Med..

[11] Cárdenas-Fernández V., Chinchilla-Minguet J.L., Castillo-Rodríguez A. (2019). Somatotype and Body Composition in Young Soccer Players According to the Playing Position and Sport Success. J. Strength Cond. Res..

[12] Katch F.I., Katch V.L. (1980). Measurement and prediction errors in body composition assessment and the search for the perfect prediction equation. Res. Q Exerc Sport..

[13] Carter J.E.L. (1980). The Heath-Carter Somatotype Method.

[14] Campa F., Matias C., Gatterer H., Toselli S., Koury J.C., Andreoli A., Melchiorri G., Sardinha L.B., Silva A.M. (2019). Classic bioelectrical impedance vector reference values for assessing body composition in male and female athletes. Int. J. Environ. Res. Public Health.

[15] Lukaski H.C., Piccoli A., Preedy V. (2012). Bioelectrical impedance vector analysis for assessment of hydration in physiological states and clinical conditions. Handbook of Anthropometry.

[16] Guo S.S., Chumlea W.C., Cockram D.B. (1996). Use of statistical methods to estimate body composition. Am. J. Clin. Nutr..

[17] Guo S.S., Chumlea W.C., Roche A.F., Heymsfield S.B., Lohman T.G. (1996). Statistical methods for the development and testing of predictive equations. Human Body Composition.

[18] Lin L. (1989). A concordance correlation coefficient to evaluate reproducibility. Biometrics.

[19] Bland J.M., Altman D.G. (1986). Statistical methods for assessing agreement between two methods of clinical measurement. Lancet.

[20] Rudnev S.G., Negasheva M.A., Godina E.Z. (2019). Assessment of the Heath-Carter somatotype in adults using bioelectrical impedance analysis. IOP Conf. Ser. J. Phys. Conf. Ser..

[21] Anisimova A.V., Godina E.Z., Nikolaev D.V., Rudnev S.G. (2016). Evaluation of the Heath-Carter somatotype revisited: New bioimpedance equations for children and adolescents. IFMBE Proc..

[22] McBride G.B. (2005). A Proposal fo Strength-of-Agreement Criteria for Lin’s Concordance Correlation Coefficient.

[23] Campa F., Silva A.M., Matias C.N., Monteiro C.P., Paoli A., Nunes J.P., Talluri J., Lukaski H.C., Toselli S. (2020). Body Water Content and Morphological Characteristics Modify Bioimpedance Vector Patterns in Volleyball, Soccer, and Rugby Players. Int J. Environ. Res. Public Health.

[24] Campa F., Silva A.M., Talluri J., Matias C.N., Badicu G., Toselli S. (2020). Somatotype and Bioimpedance Vector Analysis: A New Target Zone for Male Athletes. Sustainability.

[25] Kim C.H., Park J.H., Kim H., Chung S., Park S.H. Modeling the human body shape in bioimpedance vector measurements. Proceedings of the 2010 Annual International Conference of the IEEE Engineering in Medicine and Biology.

[26] Campa F., Matias C.N., Marini E., Heymsfield S.B., Toselli S., Sardinha L.B., Silva A.M. (2020). Identifying athlete body-fluid changes during a competitive season with bioelectrical impedance vector analysis. Int. J. Sports Physiol. Perform..

[27] Marini E., Campa F., Buffa R., Stagi S., Matias C.N., Toselli S., Sardinha L.B., Silva A.M. (2020). Phase angle and bioelectrical impedance vector analysis in the evaluation of body composition in athletes. Clin. Nutr..

[28] Piccoli A., Pastori G., Codognotto M., Paoli A. (2007). Equivalence of information from single frequency v. bioimpedance spectroscopy in bodybuilders. Br. J. Nutr..

[29] Rakovi’c A., Savanovi´c V., Stankovi´c D., Pavlovi´c R., Simeonov A., Petkovi´c E. (2015). Analysis of the elite athletes’ somatotypes. Acta Kinesiol..

[30] Sánchez Muñoz C., Muros J.J., López Belmonte Ó., Zabala M. (2020). Anthropometric characteristics, body composition and somatotype of elite male young runners. Int. J. Environ. Res. Public Health.

[31] Choudhary S., Singh S., Singh I., Varte L.R., Sahani R., Rawat S. (2019). Somatotypes of Indian Athletes of Different Sports. Online J. Health Allied Sci..

[32] Silva A.M., Matias C.N., Nunes C.L., Santos D.A., Marini E., Lukaski H.C., Sardinha L.B. (2019). Lack of agreement of in vivo raw bioimpedance measurements obtained from two single and multi-frequency bioelectrical impedance devices. Eur. J. Clin. Nutr..

[33] Nikolaidis P.T., Vassilios Karydis N. (2011). Physique and body composition in soccer players across adolescence. Asian J. Sports Med..

